# Gate-controlled amplifiable ultraviolet AlGaN/GaN high-electron-mobility phototransistor

**DOI:** 10.1038/s41598-021-86575-7

**Published:** 2021-03-30

**Authors:** Seung-Hye Baek, Gun-Woo Lee, Chu-Young Cho, Sung-Nam Lee

**Affiliations:** 1 Department of Nano and Semiconductor Engineering, Korea Polytechnic University, Siheung, 15073 Republic of Korea; 2 Nanodevices Lab., Korea Advanced Nano Fab Center, Suwon, Gyeonggi-do 16229 Republic of Korea

**Keywords:** Materials for devices, Electrical and electronic engineering, Electronics, photonics and device physics

## Abstract

Gate-controlled amplifiable ultraviolet phototransistors have been demonstrated using AlGaN/GaN high-electron-mobility transistors (HEMTs) with very thin AlGaN barriers. In the AlGaN/GaN HEMTs, the dark current between the source and drain increases with increasing thickness of the AlGaN barrier from 10 to 30 nm owing to the increase in piezoelectric polarization-induced two-dimensional electron gas (2-DEG). However, the photocurrent of the AlGaN/GaN HEMT decreases with increasing thickness of the AlGaN barrier under ultraviolet exposure conditions. It can be observed that a thicker AlGaN barrier exhibits a much higher 2-DEG than the photogenerated carriers at the interface between AlGaN and GaN. In addition, regardless of the AlGaN barrier thickness, the source–drain dark current increases as the gate bias increases from − 1.0 to + 1.0 V. However, the photocurrent of the phototransistor with the 30 nm thick AlGaN barrier was not affected by the gate bias, whereas that of the phototransistor with 10 nm thick AlGaN barrier was amplified from reduction of the gate bias. From these results, we suggest that by controlling the gate bias, a thin AlGaN barrier can amplify/attenuate the photocurrent of the AlGaN/GaN HEMT-based phototransistor.

## Introduction

The class of III-nitride semiconductors are very promising materials for manufacturing high-performance optoelectronic devices such as light-emitting diodes, laser diodes and photodetectors (PDs) because of their wide band gap energies and highly stable chemical/physical properties^1–5^. In particular, there has been considerable interest in GaN-based devices like ultraviolet (UV) PDs^6–8^. because of their wide applications in military and commercial fields such as flame monitoring, space ozone monitoring, etc^9,10^. While comparing with other UV detectors based on photoconductors, AlGaN/GaN heterostructure HEMTs can provide high internal gain, favored by highly conductive two-dimensional electron gas (2-DEG) channel at the heterostructure interface due to the built-in electric field induced by strong piezoelectric and spontaneous polarizations^11–17^. In the AlGaN/GaN high-electron-mobility transistors (HEMTs), the channel conductivity is increased by UV exposure, resulting in a significant change in drain current. Therefore, a few research groups have demonstrated very high responsivities (up to ~ 10^7^ A/W) in AlGaN/GaN heterostructure-based devices^18–21^. In the UV detection mechanism of AlGaN/GaN HEMT structure, it is known that the surface charge states and the absorption at the GaN channel layer increase 2-DEG channel conductance^22–24^. In particular, it has been reported that the optical gain is significantly dominated by absorption in the GaN channel layer rather than the AlGaN barrier^25^. Moreover, to further improve photoresponsivity of AlGaN/GaN heterostructure, nanostructures such as ZnO nanorods or graphene are introduced between the source and drain electrodes^26,27^.

In the control of the source–drain current (I_DS_), AlGaN/GaN HEMT structure can be significantly affected by the AlGaN barrier and gate bias^24,25^. In particular, the thickness and composition of the AlGaN barrier are major factors in the formation of 2-DEG, which can improve the channel conductance^24^. It is known that the gate bias can control the drain current due to carrier accumulation/depletion in the channel region^24^. However, the effect of gate bias on photocurrent of AlGaN/GaN HEMTs with different AlGaN barriers has not yet been analysed. Therefore, we investigated the effect of AlGaN barrier on the electrical and optical properties of AlGaN/GaN HEMT-based phototransistor by applying the gate bias. Furthermore, we demonstrated amplifiable photocurrent of AlGaN/GaN phototransistor with very thin AlGaN barrier by controlling the gate bias.

## Structure and optical properties of AlGaN/GaN HEMTs with different AlGaN barrier thickness

**Figure 1:**
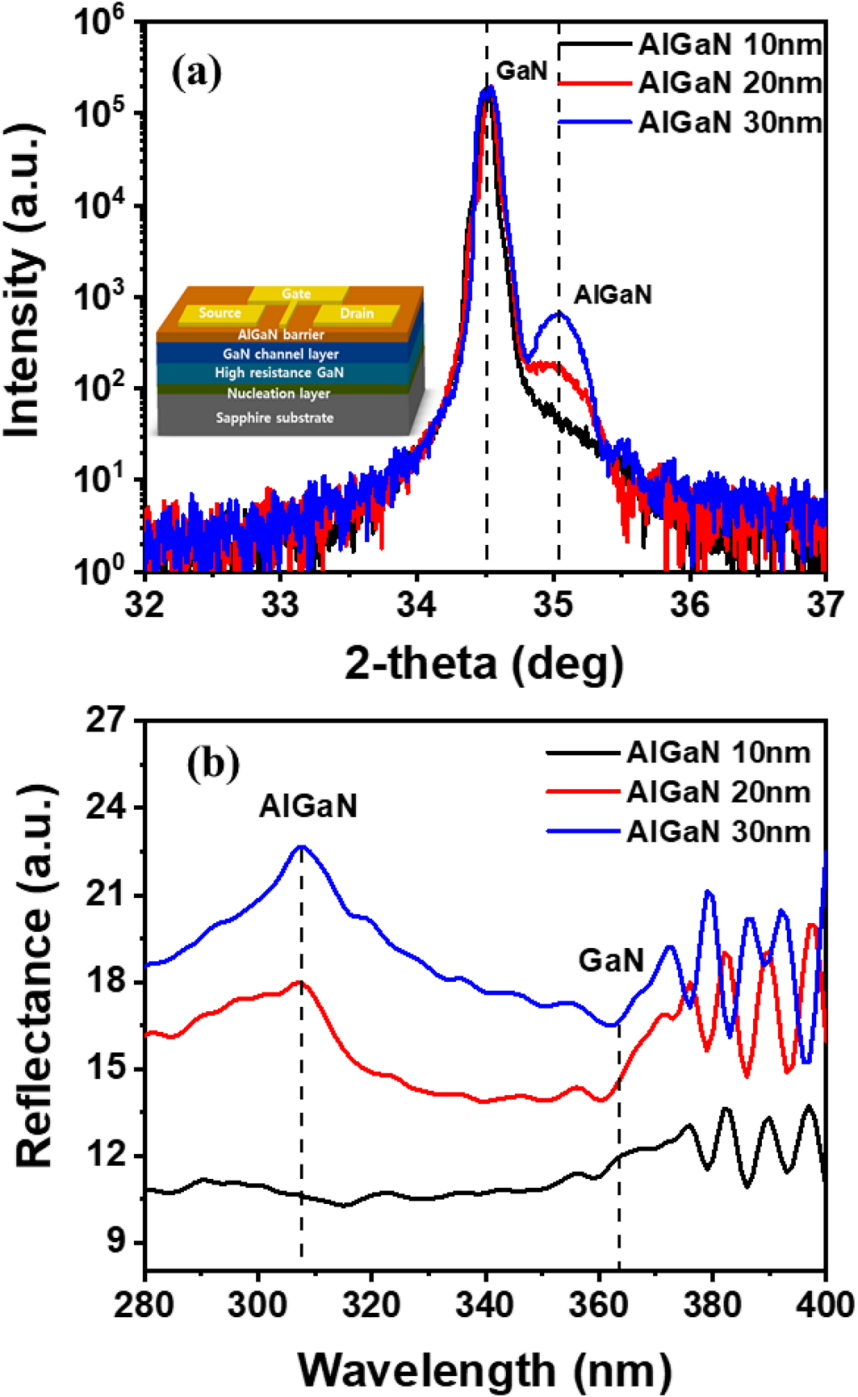
Structure and optical properties of AlGaN/GaN HEMTs with different AlGaN barrier thicknesses characterized using X-ray (002) ω/2θ scans (**a**) and optical reflectance analysis (**b**). The inset shows a schematic of the AlGaN/GaN HEMT with the source, drain, and gate electrodes.

Figure 1a shows the high-resolution X-ray diffraction (HR-XRD) ω/2θ scans of the AlGaN/GaN HEMTs with different AlGaN barrier thicknesses. It exhibits one peak each at 34.51° and 35.04° which corresponds to the (002) GaN film and AlGaN barrier layer, respectively. The aluminum compositions of the AlGaN barrier layers were calculated using Bragg’s law (nλ = 2dsinθ, where n is an integer representing the order of the diffraction peaks, λ is the wavelength (0.154 nm) of X-ray CuKα, and θ is the diffraction angle) and Vegard’s law^27–29^. These results reported that the three samples had the same aluminum composition of 30% in the AlGaN/GaN heterostructures. In addition, the XRD intensity of the Al_0.3_Ga_0.7_N barrier increased proportionally with thickness and the interference fringe was developed more clearly as the thickness of the AlGaN barrier increased. It indicates that the interface quality between AlGaN and GaN is improved with an increase in the thickness of AlGaN barrier, which can improve the mobility of 2-DEG. Figure 1b shows the reflectance spectra of AlGaN/GaN HEMT structure with different AlGaN barrier thicknesses. The peak before the start of the oscillation can be readily observed and the band gap energies of AlGaN barrier and GaN channel layer were measured to be 4.03 eV and 3.41 eV, respectively, which is consistent with the values of AlGaN and GaN obtained from HR-XRD shown in Fig. 1a. In addition, the target thickness of the AlGaN barriers is observed to be nearly the same as that calculated using Fabry–Perot oscillations ranging from 310 to 360 nm^30^.

## Effect of AlGaN barrier thickness on dark current and photocurrent between source and drain of AlGaN/GaN HEMTs

**Figure 2:**
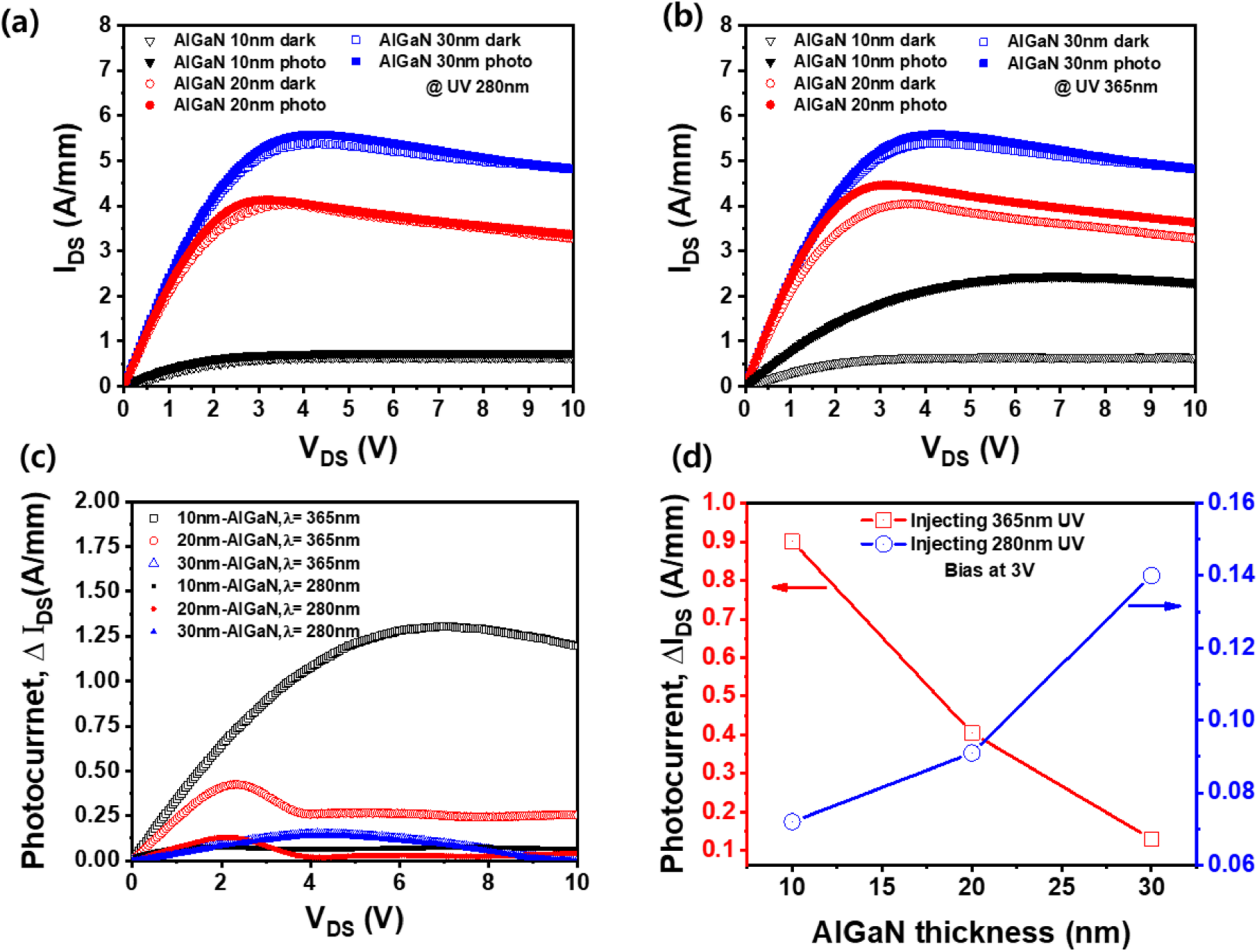
I–V characteristics of AlGaN/GaN HEMTs with different AlGaN barrier thicknesses under dark and UV illuminations using 280 nm (**a**) and 365 nm (**b**) wavelengths as exposure light sources. (**c**) Photocurrent (ΔI_DS_) versus source–drain voltages of HEMTs with different AlGaN barrier thicknesses using 280 nm and 365 nm UV light. (**d**) Photocurrent between source and drain as a function of AlGaN barrier thickness at 3.0 V drain voltage.

Figure 2a,b shows the source–drain dark current (I_DS_) and photocurrent (ΔI_DS_) of AlGaN/GaN HEMTs with different AlGaN barrier thicknesses in dark and UV exposures at 280 nm and 365 nm, respectively. In the dark, as the thickness of AlGaN barrier increased, the source–drain current increased from 0.45 to 4.06 A/mm. In general, it is known that a higher Al composition and a thicker AlGaN barrier increases the 2-DEG channel density formed at the interface between the AlGaN barrier and the GaN channel layer due to the strong piezoelectric polarization effect resulting in high source–drain current flow^11–16,31^. The source–drain photocurrents of AlGaN/GaN HEMT were increased by exposure of 280 nm and 365 nm UV light compared to the dark state. It can be explained by the increase of channel conductance due to the photogenerating carrier of AlGaN barrier and GaN channel layer^25^. In addition, it can be seen that the increase of source–drain photocurrent by UV exposure is obtained at the maximum pinch-off voltage just before the onset of the conventional saturation region shown in Fig. 2c. The source–drain photocurrent and dark current are reduced by self-heating effect at the drain bias at a voltage higher than the pinch-off voltage. This is because the induced open circuit photovoltage decreases with increasing temperature, which is well known in solar cell theory^32^.

Figure 2d shows the photocurrent of AlGaN/GaN HEMT with different AlGaN barrier thicknesses using 280 and 365 nm UV light. The photocurrent was calculated using the equation of ΔI_DS_ = I_DS(UV)_ − I_DS(dark)_, where I_DS(UV)_ and I_DS(dark)_ are the UV illumination current and dark current obtained between the source and drain, respectively. As the AlGaN barrier thickness increased from 10 to 30 nm, 280 nm-UV induced photocurrent increased from 0.07 A/mm to 0.14 A/mm, whereas 365 nm-UV induced photocurrent decreased from 0.93 A/mm to 0.14 A/mm. Since 280 nm-UV light is mostly absorbed in the AlGaN barrier region, the photocurrent of AlGaN/GaN HEMT is proportional to the thickness of AlGaN barrier layer. However, since 365 nm UV light has lower energy than the band gap of the AlGaN barrier layer, 365 nm UV light is mainly adsorbed in the GaN channel rather than the AlGaN barrier. Moreover, it has been reported that the photoexcitation carrier of the GaN film is higher than that of the AlGaN film^24^. Therefore, it is expected that as the thickness of the AlGaN barrier layer decreases, 365 nm-UV absorption of the GaN film increases and the photocurrent increases. In addition, it is found that the photocurrent obtained from 365 nm-UV exposure is much higher than 280 nm-UV light. In particular, 365 nm UV-induced photocurrent (0.93 A/mm) is 13.3 times higher than 280 nm UV-induced photocurrent (0.07 A/mm) in the AlGaN/GaN HEMT with a 10 nm thick AlGaN barrier under the applied drain voltage (V_DS_) of 3.0 V. The 2-DEG density (1.05 × 10^13^/cm^2^) of AlGaN/GaN HEMT with a 30 nm thick AlGaN barrier is much higher than that of a 10 nm thick AlGaN barrier (8.59 × 10^12^/cm^2^), leading to the higher dark current (I_DS(dark)_) of HEMT with a 30 nm thick AlGaN barrier than a 10 nm thick AlGaN barrier. Moreover, since the transmission of UV light through the AlGaN barrier is reduced by increasing the thickness of AlGaN barrier, the photogenerated carriers of HEMTs with 10 nm AlGaN barrier are higher than those of HEMTs with 30 nm AlGaN barrier. Therefore, it is believed that the photocurrent of HEMT with a 10 nm thick AlGaN barrier is higher than that of a 30 nm thick AlGaN barrier owing to the higher photogenerated carrier than the low dark current formed by the relative low 2-DEG in HEMT with a 10 nm thick AlGaN barrier.

## UV wavelength dependent photoresponsivity of AlGaN/GaN HEMTs with different thicknesses of AlGaN barriers

**Figure 3:**
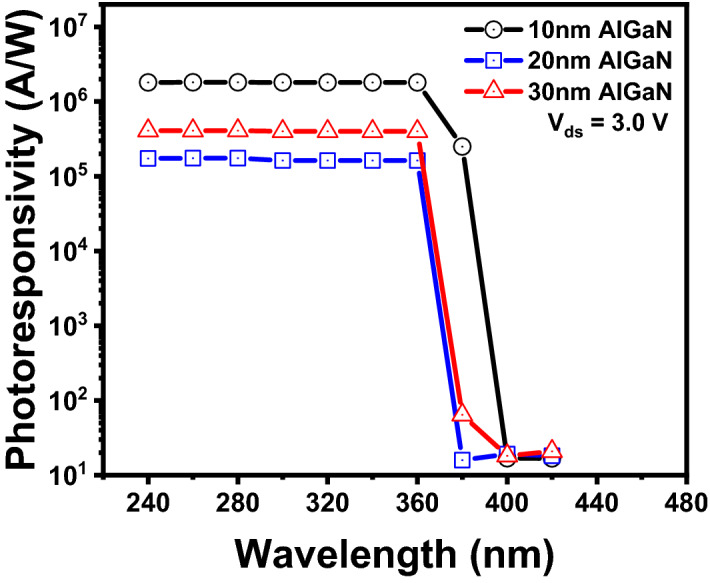
Photocurrents between the source and drain of AlGaN/GaN HEMTs with different AlGaN barrier thicknesses as functions of exposure light wavelengths.

To investigate the absorption regions of the AlGaN barrier and GaN buffer layers, AlGaN/GaN phototransistors were irradiated with 240–420 nm UV light using a UV monochromator with a Xenon lamp source covering the band gap energies for the AlGaN and GaN regions. The power at each wavelength was maintained the same to avoid strong gain dependence on the input optical power. Figure 3 shows the photoresponsivity of AlGaN/GaN HEMTs with different AlGaN barrier thicknesses as a function of excitation wavelength. The photoresponsivity (R) and gain (g) of photodetector can be calculated by following equations^19,21^

1 $${\text{R }} = ({\text{I}}_{{{\text{UV}}}} - {\text{ I}}_{{{\text{dark}}}} )/{\text{P}}_{{\text{o}}} = {\text{I}}_{{\text{ph}}} /{\text{P}}_{{\text{o}}} = {\text{ gqLG}}_{{\text{L}}} /{\text{P}}_{{\text{o}}} ,$$

where I_ph_, P_o_, g, L, and G_L_ are the photocurrent of photodetector, the optical power of exposure light, device gain, sensing space and the generation rate of electron–hole pair, respectively. Although the dark current (I_dark_) of 10 nm-thick AlGaN barrier HEMT is much lower than that of 30 nm-thick AlGaN barrier HEMT, the photocurrent of 10 nm-thick AlGaN barrier HEMT is much higher than that of 30 nm-thick AlGaN barrier HEMT as shown in Fig. 2b,c. Thus, the maximum photoresponsivity (1.44 × 10^6^ A/W) of AlGaN/GaN HEMT is obtained using 10 nm thick AlGaN barrier. All samples had a sharp transition near the GaN cutoff wavelength of 360 nm, while no significant change was observed at the AlGaN cutoff wavelength near 300 nm as shown in Fig. 3. In addition, the AlGaN/GaN HEMTs with 20 nm and 30 nm thick AlGaN barriers show a very slight decrease of photoresponsivity near the band gap (~ 300 nm) of AlGaN barrier. In addition, from Eq. (1), it can be seen that the photoconductive gain is proportional to the photoresposivity^21,33^. Thus, the photoconductive gain of HEMT with 10 nm thick AlGaN barrier is higher than that of HEMT with 30 nm thick AlGaN barrier due to higher UV photocurrent (I_ph_). It is another clear evidence that the photoconductive gain from absorption is dominant in the GaN region rather than the AlGaN barrier layer and can be increased using a thinner AlGaN barrier^24^. In addition, since the photoresponsivity is proportional to the generation rate of electron–hole pairs shown in Eq. (1) ^21^, the photogeneration rate of the high photoresponsivity HEMTs with 10 nm AlGaN barrier is expected to be higher than that of the low photoresponsivity HEMTs with 30 nm AlGaN barrier. It means that HEMTs with 10 nm AlGaN barrier can have a fast response to UV exposure.

## Gate bias dependence of photocurrent in AlGaN/GaN HEMTs with different AlGaN barrier thicknesses

**Figure 4:**
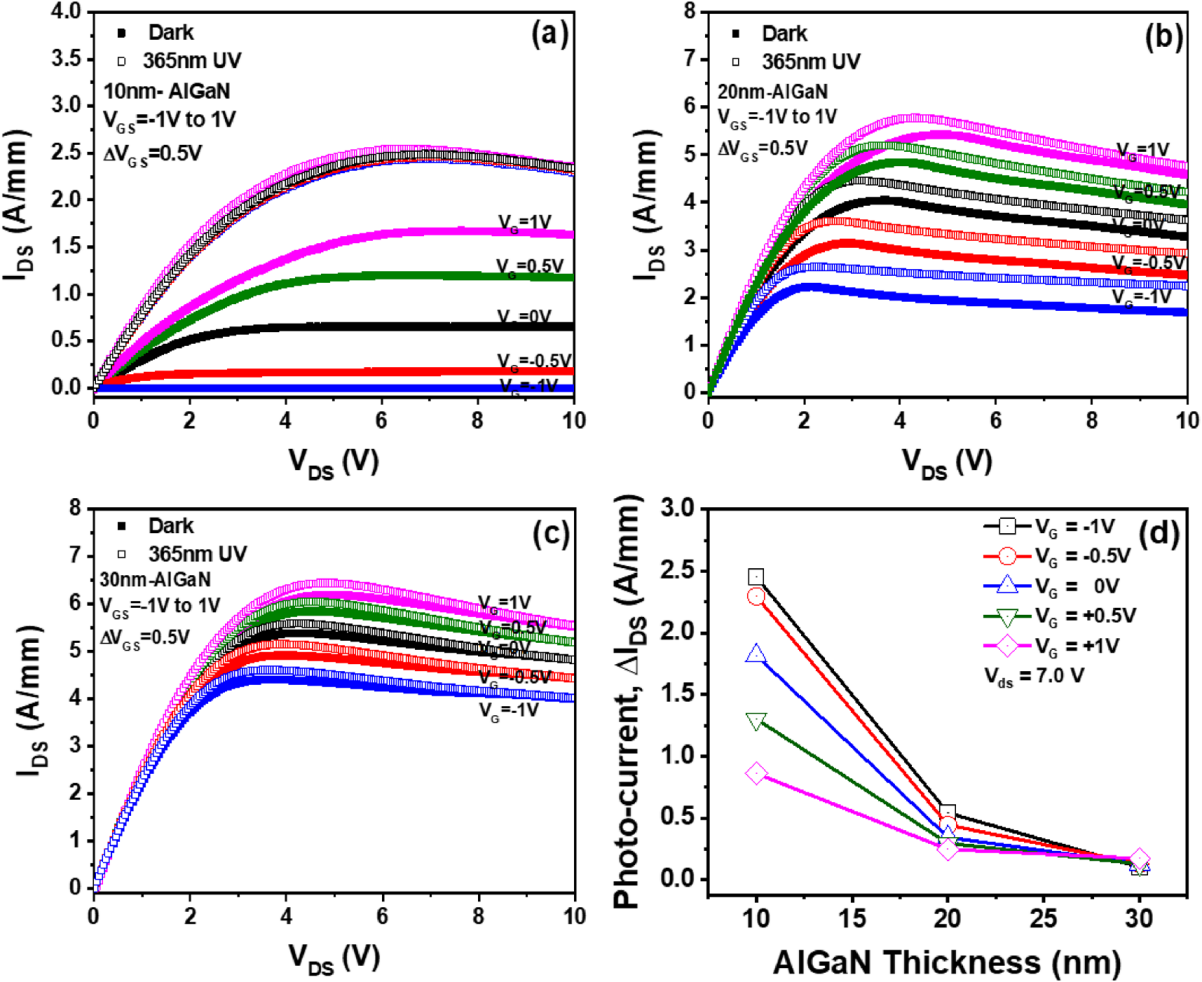
I_DS_–V_DS_ characteristics of AlGaN/GaN HEMTs with 10 nm (**a**), 20 nm (**b**), and 30 nm (**c**) thick AlGaN barriers for dark (close square) and UV (open square) illumination conditions. (**d**) Photocurrent characteristics of HEMTs with different gate biases as functions of AlGaN barrier thicknesses.

We analyzed the effect of gate bias on the I_DS_–V_DS_ characteristics of AlGaN/GaN HEMTs with 10 nm, 20 nm, and 30 nm thick AlGaN barriers in the dark state and UV (365 nm) illumination conditions as shown Fig. 4a–c, respectively. In the dark state, regardless of the thickness of AlGaN barrier, the drain current increased as the gate bias increased from − 1.0 V to + 1.0 V. This is typical electrical behavior in AlGaN/GaN HEMT due to the increase of electron in the 2-DEG channel region by increasing the positive gate bias field^34^. However, at constant gate bias, the drain current of AlGaN/GaN HEMT increased with AlGaN barrier thickness due to the increase of piezoelectric polarization-induced 2-DEG formed at the AlGaN/GaN interface. As a result, it can be seen thought that higher current flow between the source and drain is achieved by applying a high gate and drain voltage in the dark state. In addition, we measured the source–drain current of three AlGaN/GaN HEMTs with different gate voltages in 365 nm UV illumination. Regardless of the thickness of AlGaN barrier and the gate bias, the source–drain currents of HEMTs were increased by 365 nm-UV exposure. Moreover, the pinch-off voltage is decreased by UV illumination. It indicates that the 2-DEG-related source–drain current of AlGaN/GaN HEMT is significantly affected by the photogenerated carrier due to the exposure of 365 nm-UV light. However, as the thickness of AlGaN barrier and the gate voltage increased, the increase rate of the photocurrent decreased as shown in Fig. 4d. In the AlGaN/GaN HEMT with 10 nm thick AlGaN barrier, the source–drain current is significantly reduced by increasing the negative gate voltage in the dark state, while the source–drain current is hardly affected by the gate bias under the UV illumination. As a result, the highest photocurrent can be achieved using 10 nm thick AlGaN barrier and gate voltage of − 1.0 V. The source–drain current of HEMT with 30 nm thick AlGaN barrier is not significantly affected by the photogenerated carrier because the 2-DEG is higher than the photogenerated carrier. However, since the HEMT with 10 nm thick AlGaN barrier had a lower 2-DEG than the photogenerated carrier, the source–drain photocurrent is significantly increased by UV illumination. Based on these results, we believe that HEMTs with low 2-DEG using very thin (~ 10 nm) AlGaN barrier and high negative bias can be effective in achieving high photocurrent because they can have higher photogenerated carrier than 2-DEG.

## Effect of AlGaN barrier thickness on gate bias-controlled amplifiable photocurrent of AlGaN/GaN HEMTs

**Figure 5:**
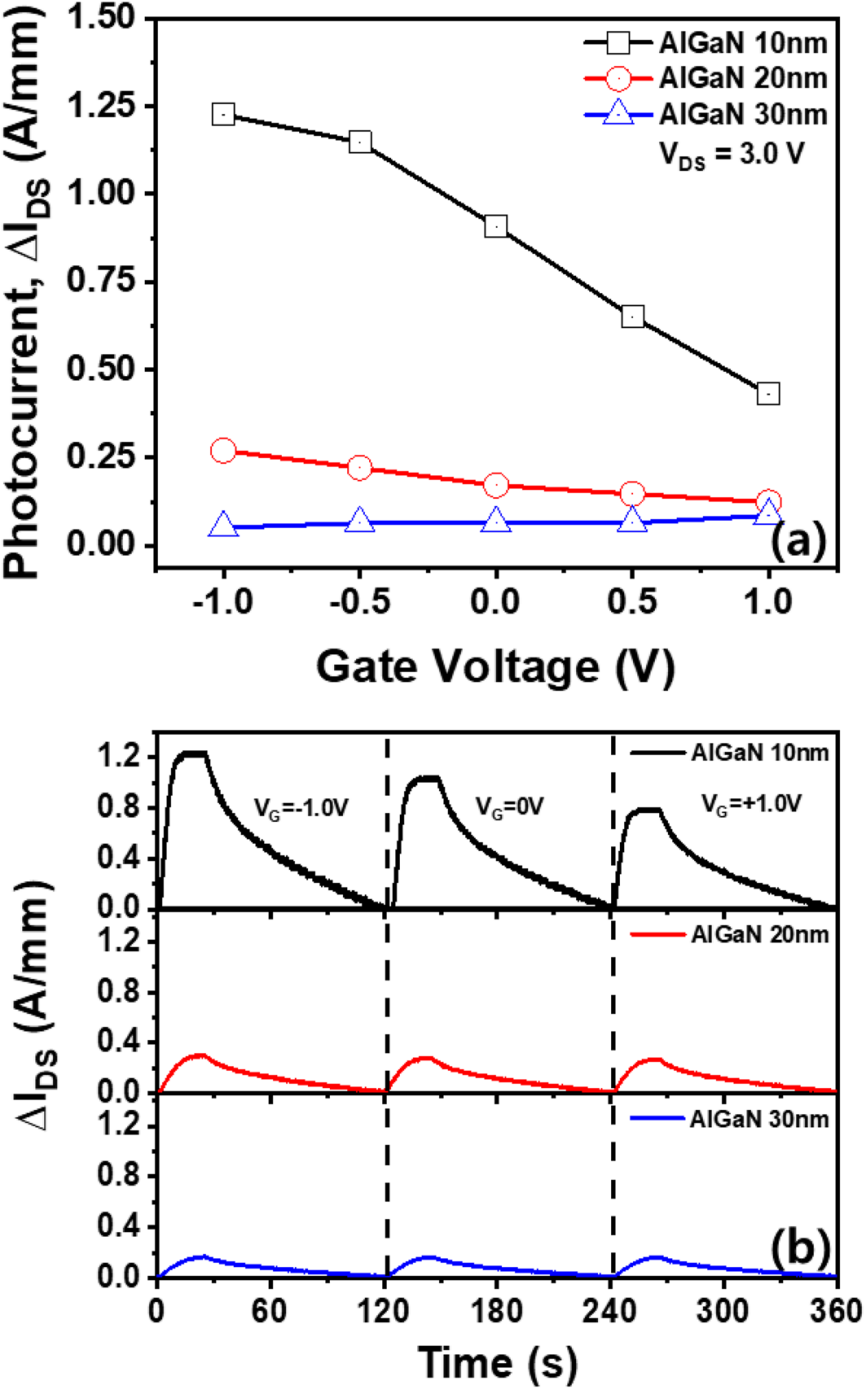
Photocurrent characteristics of HEMTs with (**a**) 10 nm, 20 nm, and 30 nm thick AlGaN barriers at the applied gate voltage of 3.0 V as a function of different AlGaN barrier thicknesses. (**b**) Time-dependent photocurrents of HEMTs with different AlGaN barriers; each duration was applied a different gate voltage (V_G_ = − 1.0 V for 0–120 s, V_G_ = 0 V for 120–240 s, and V_G_ = + 1.0 V for 240–360 s).

Figure 5a shows the photocurrent of AlGaN/GaN HEMTs with different AlGaN barrier thicknesses as a function of gate voltage. It indicates that the photocurrent reduces with an increase in the thickness of the AlGaN barrier. In addition, the photocurrent of HEMT with a 30 nm thick AlGaN barrier is hardly affected by the gate bias, whereas the photocurrent of HEMT with a 10 nm thick AlGaN barrier increased from 0.39 to 1.22 A/mm as the gate bias decreased from + 1.0 V to − 1.0 V. In other words, it can be seen that a photocurrent of HEMT with a 10 nm thick AlGaN barrier can be amplified/reduced by applying positive/negative gate voltages, respectively. Thus, we measured the time-dependent photocurrent of AlGaN/GaN HEMTs with different AlGaN barrier thicknesses by turning the UV light on/off and controlling the gate bias from − 1.0 V to + 1.0 V shown in Fig. 5b. Regardless of AlGaN barrier thickness, the AlGaN/GaN HEMTs were operated well periodically by turning the UV light on/off and controlling the gate bias, indicating that all HEMTs have good and reliable properties. In addition, HEMT with a 30 nm thick AlGaN barrier has similar low photocurrents at different gate biases, whereas HEMTs with thin (≤ 20 nm) AlGaN barriers exhibit an amplification/reduction of gate-bias induced photocurrent. In particular, it can be seen that an HEMT having a 10 nm thick AlGaN barrier can dramatically control the photocurrent by controlling the gate bias. Moreover, it is observed that the response time of AlGaN/GaN HEMT becomes faster as the thickness of AlGaN barrier decreases. Since the 10 nm thick AlGaN barrier has a relatively lower 2-DEG than the 30 nm thick AlGaN barrier, the photogenerated carrier can easily exceed 2-DEG in the 10 nm thick AlGaN barrier, resulting in a fast rising time of photocurrent. In addition, the higher Al composition and thicker AlGaN barrier grown on GaN channel layer may have more crystal defects forming impurity-related deep levels such as misfit dislocations and point defects that can play a role in absorbing UV ligth at the top AlGaN barrier^36^. As a result, these crystal defects can delay the formation of the photogenerated carriers in the AlGaN barrier and the GaN channel layer, resulting in a low response time of the AlGaN/GaN HEMT with a 30 nm thick AlGaN barrier layer. Based on these results, we suggest that the photocurrent of AlGaN/GaN HEMT with a thin AlGaN barrier can be amplified/reduced by controlling the gate bias.

## Schematic band biagram of AlGaN/GaN HEMTs with different AlGaN barrier thicknesses by applied gate bias and UV illumination

**Figure 6:**
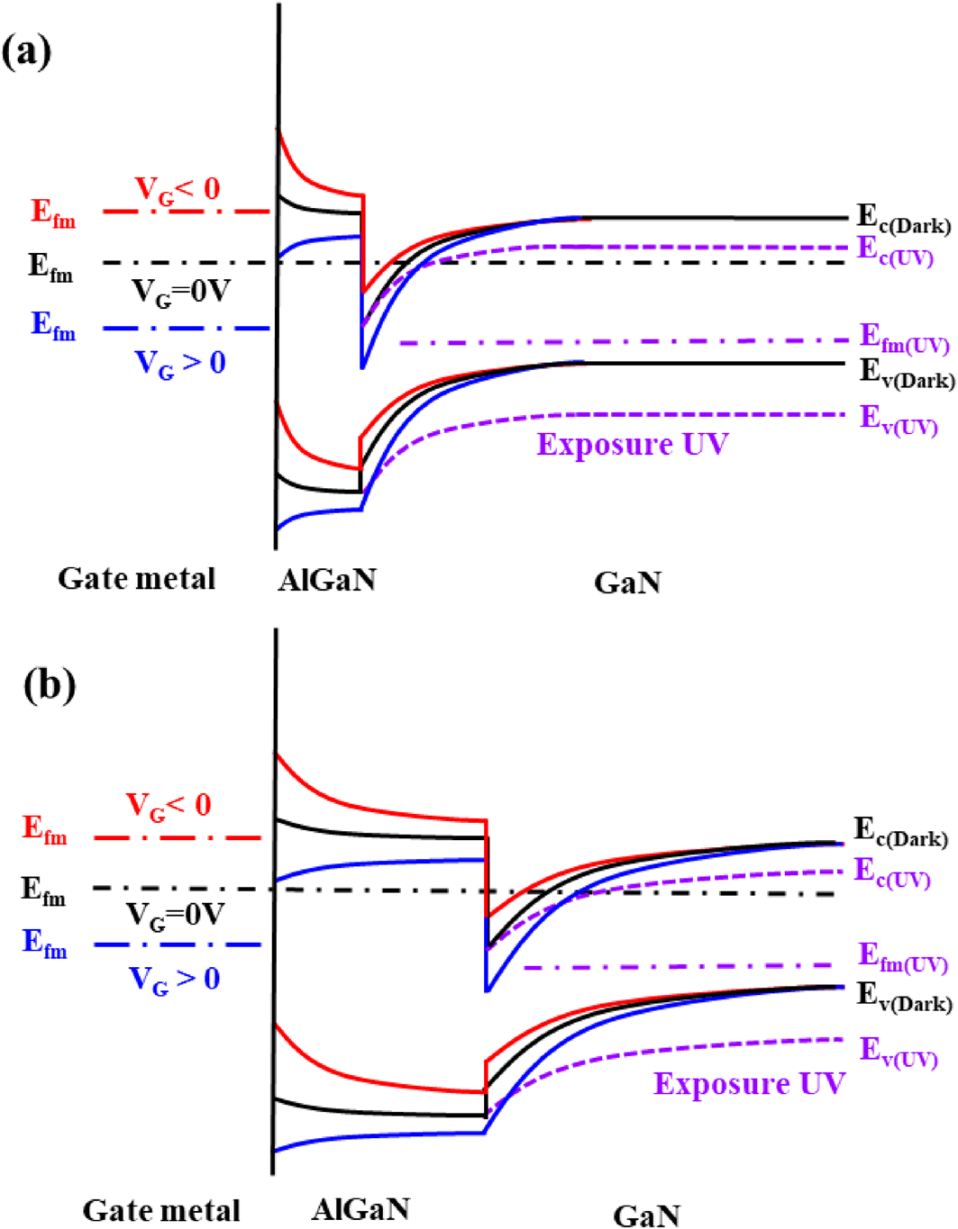
Schematic band diagrams of AlGaN/GaN HEMTs with 10 nm (**a**) and 30 nm (**b**) thick AlGaN barriers by applying zero (black line), positive (blue line), and negative (red line) gate biases. The solid and dotted lines depict bands with dark and UV illuminations, respectively.

Figure 6 shows the band diagrams of AlGaN/GaN HEMTs with 10 nm (a) and 30 nm thick (b) AlGaN barriers by applying negative/positive gate biases and exposing them to UV light. The interface between the AlGaN and GaN films has strong band bending due to polarization properties. As a result, the 2-DEG channel increases with the AlGaN barrier thickness because of the increase of strain-induced polarization effect at the interface of AlGaN/GaN. At gate zero bias (V_applied_ = 0 V), the Fermi level of the gate metal is same as the Fermi level of AlGaN/GaN, indicating that the HEMT device is in equilibrium condition^35^. When UV light is injected, excess holes generated on GaN buffer layer accumulate at the interface of GaN/substrate, causing the bands to bend down and a positive back-gate bias maintained by the photocurrent, which increases 2-DEG^25^. In particular, since the 2-DEG channel of HEMT with a 30 nm thick AlGaN barrier is much wider than a 10 nm thick AlGaN barrier, the ratio of photogenerated carriers by bending down in the 30 nm thick AlGaN barrier is relatively lower than that in the 10 nm thick AlGaN barrier shown in Fig. 6a,b. Therefore, it can be seen that the photocurrent of AlGaN/GaN HEMT decreases with increasing the thickness of AlGaN barrier, which is consistent with Fig. 4d. In addition, when the gate voltage is applied by negative (red line) and positive (blue line) bias, the Fermi level of gate metal moves up and down, respectively as shown in Fig. 6. In this case, the energy band between the metal and the AlGaN film goes upward and downward, resulting in the decrease and increase of 2DEG channel, respectively. In particular, due to the downward bending effect by UV irradiation, the increase rate of 2-DEG channel is higher in the 10 nm thick AlGaN barrier than the 30 nm thick AlGaN barrier. Therefore, we suggest that the 10 nm thick AlGaN barrier HEMTs with relatively low 2-DEG can amplify/reduce the photocurrent more easily than the 30 nm thick AlGaN barrier with high 2-DEG by controlling the negative/positive gate bias, respectively.

## Conclusions

We have achieved a gate-controlled amplifiable ultraviolet AlGaN/GaN HEMT-based phototransistor using a 10 nm thick AlGaN barrier. As the thickness of the AlGaN barrier increased from 10 to 30 nm, the dark current between the source and drain increased due to the increase of piezoelectric polarization-induced 2-DEG. However, the photocurrent of the AlGaN/GaN HEMT decreases with increasing the thickness of the AlGaN barrier under UV exposure conditions. In addition, as the gate bias increased from − 1.0 to + 1.0 V, the source–drain dark current increased, which is a typical behavior of HEMT. However, the photocurrent of HEMT with a 30 nm thick AlGaN barrier was barely affected by the gate bias, whereas that of the HEMT with the 10 nm thick AlGaN barrier was significantly amplified by reducing the gate bias. Based on these results, we suggest that HEMTs with a low 2-DEG channel can more easily amplify/reduce photocurrents than HEMTs with high 2-DEG by controlling gate bias.

## Methods

The AlGaN/GaN heterostructure of this work was grown on 4-inch *c*-plane (0001) sapphire substrates by metalorganic chemical vapor deposition. Trimethylgallium, trimethylaluminum were used as group III precursors and ammonia (NH_3_) was used as a nitrogen source. The epitaxial structure consists of a high-resistance (HR) GaN buffer, a GaN channel layer, and an AlGaN barrier layer, as shown in inset of Fig. 1a. After growing the 30 nm thick GaN nucleation layer at 480 °C, the 2.0 μm thick HR-GaN buffer layer was grown at 1000 °C. HR-GaN buffer layer can be achieved by carbon incorporation because carbon contributes to the compensation of free carrier. Subsequently, a 150 nm thick GaN channel layer was grown with high crystalline quality, considering the 2-DEG characteristics and wafer uniformity. Finally, 10, 20, and 30 nm thick AlGaN barrier layers with an Al composition of 30% were grown on the GaN channel layers to investigate the effect of the thickness of AlGaN barrier. The grown epitaxial wafer was fabricated with a Hall bar structure and HEMTs for electrical evaluation. All device patterns were defined by optical lithography. Mesa isolation was performed with an inductive coupled plasma of BCl_3_/Cl_2_ gas mixture. The source and drain electrodes were formed with the metal stack of Ti/Al/Ni/Au (20/100/25/50 nm) by e-beam evaporation and then annealed at 850 °C in N_2_ ambient. Finally, Ni/Au metals (30/270 nm) were evaporated for gate metallization. The gates were centered within the drain-source. Overlay metallization on ohmic contacts and the measurement pads were also deposited during gate formation. The devices had a gate width of 100 µm and a gate length of 2.0 µm. The source–drain spacing was 8 µm and the sensing area was approximately 600 µm^2^.

We analyzed the crystal and optical properties of AlGaN/GaN heterostructured phototransistor with different AlGaN barrier thicknesses using RIGAKU DMAX 2200 high resolution X-ray diffraction (HR-XRD) and THERMO SCIENTIFIC EVOLUTION 300 UV–visible spectroscopy, respectively. To evaluate the electrical properties, current–voltage (I–V) measurements of dark current and photocurrent were performed using the HP4155 parameter analyzer. For the UV illumination, a Xenon lamp with an exposure power of 17 μW/cm^2^ at the wavelength of 360 nm was used as an excitation light source to observe the photocurrent.
